# Healthcare utilisation in general practice and hospitals in the year preceding a diagnosis of cancer recurrence or second primary cancer: a population-based register study

**DOI:** 10.1186/s12913-019-4757-y

**Published:** 2019-12-05

**Authors:** Linda Aagaard Rasmussen, Henry Jensen, Line Flytkjær Virgilsen, Alina Zalounina Falborg, Henrik Møller, Peter Vedsted

**Affiliations:** 1 Research Unit for General Practice, Aarhus, Denmark; 2 Research Centre for Cancer Diagnosis in Primary Care (CaP), Department of Public Health, Aarhus University, Aarhus, Denmark; 3 Danish Clinical Registries (RKKP), Aarhus, Denmark

**Keywords:** Delivery of healthcare, Cancer survivors, Recurrence, Second primary neoplasms, Primary health care, Care transition

## Abstract

**Background:**

The organisation of cancer follow-up is under scrutiny in many countries, and general practice is suggested to become more involved. A central focus is timely detection of recurring previous cancer and new second primary cancer. More knowledge on the patient pathway before cancer recurrence and second primary cancer is warranted to ensure the best possible organisation of follow-up. We aimed to describe the healthcare utilisation in the year preceding a diagnosis of cancer recurrence or second primary cancer.

**Methods:**

This nationwide register study comprises patients diagnosed with bladder, breast, colorectal, endometrial, lung, malignant melanoma and ovarian cancer in Denmark in 2008–2016. The frequency of healthcare contacts during the 12 months preceding a cancer recurrence or second primary cancer was estimated and compared to the frequency of cancer survivors in cancer remission. The main analyses were stratified on sex and healthcare setting. Furthermore, two sub-analyses were stratified on 1) sex, healthcare setting and age group and on 2) sex, healthcare setting and comorbidity status.

**Results:**

The study population consisted of 7832 patients with recurrence and 2703 patients with second primary cancer. On average, the patients were in contact with general practice one time per month in the 12th month preceding a new cancer diagnosis (recurrence or second primary cancer). Increasing contact rates were seen from 7 months before diagnosis in general practice and from 12 months before diagnosis in hospitals. This pattern was more pronounced in patients with cancer recurrence, younger patients and patients with no comorbidity. For instance, the contact rate ratios for hospital contacts in non-comorbid women with recurrence demonstrated 30% more contacts in the 12th month before recurrence and 127% more contacts in the 2nd month before recurrence.

**Conclusions:**

The results show that cancer survivors are already seen in general practice on a regular basis. The increasing contact rates before a diagnosis of cancer recurrence or second primary cancer indicate that a window of opportunity exists for more timely diagnosis; this is seen in both general practice and in hospitals. Thus, cancer survivors may benefit from improvements in the organisation of cancer follow-up.

## Background

Cancer survivorship has received increasing focus worldwide in recent years. Detection of cancer recurrence (CR) and second primary cancer (SPC) is one of the main foci in cancer follow-up programmes [1]. Studies report CR proportions of 4–29%, depending on cancer type, cancer stage, age and follow-up time [2–5]. Moreover, cancer survivors have a higher risk of getting a new primary cancer compared to the background population without a cancer history. A relative risk of 17% in women and 11% in men has been reported [6]. Treatment options for CR have improved, and the focus on timely detection of CR and SPC may lead to better prognosis [7–9], as shown for some primary cancers [10, 11].

Follow-up programmes after cancer treatment are currently being re-organised in many countries towards higher involvement of general practice [1, 12, 13]. Conflicting views among patients and general practitioners (GPs) have been reported on the GP’s involvement in follow-up cancer care and the derived effect on diagnostic delays in subsequent cancers [14–20]. However, the evidence is sparse on the GP’s role in detecting CR and SPC, and where and when cancer survivors seek healthcare prior to a diagnosis of a subsequent cancer.

Studies focusing on first-time cancer diagnosis have shown higher diagnostic activity and increasing numbers of contacts to general practice and hospitals for up to one year before the diagnosis [21, 22]. The authors of these studies concluded that the increased pre-diagnostic healthcare activity indicated a window of opportunity for earlier diagnosis. Recently, a small-scale Norwegian study [23] found the mean number of days to be 39 (standard deviation: 35) from the first diagnostic workup initiated on CR suspicion until a CR diagnosis. More knowledge of cancer survivors’ patterns of overall healthcare utilisation leading up to CR and SPC could identify potential areas for improvement to shorten the time to diagnosis. This new knowledge could also inform policy-makers and help optimise the future organisation of cancer follow-up programmes, including well-organised diagnostic pathways and more involvement of general practice.

The aim of this study was to investigate healthcare utilisation in the year preceding a diagnosis of CR or SPC compared to the healthcare utilisation in patients in continued cancer remission by exploring the healthcare setting (general practice and hospitals) and selected patient factors (age and comorbidity).

## Methods

### Study design and setting

The study was a population-based matched cohort study using prospectively recorded data from Danish national health registries. The study was set in Denmark, where the tax-funded healthcare system provides free access for all citizens to general practice and hospital services. More than 98% of the Danish population are registered with a specific general practice [24].

### Study population

We identified all first-time cancer patients diagnosed in 2008–2016 with the following cancer diagnoses coded according to the International Classification of Diseases, 10th revision (ICD-10) in the Danish Cancer Registry [25]: colorectal (C18, C20), lung (C34), malignant melanoma (C43), breast (C50), endometrial (C54), ovarian (C56) and bladder cancer (C67). Patients aged ≥18 years at the time of the primary cancer diagnosis and in complete cancer remission after curative intent cancer treatment were eligible for inclusion. The study population was subsequently divided into three groups: patients who developed CR, patients who were diagnosed with an SPC and patients in remission. Patients were followed from the first day in remission until the date of the first of the following events: a subsequent cancer event (either CR or SPC), death, emigration or 31 December 2016, whichever came first. We excluded patients not listed with a general practice and patients who had lived outside Denmark at any point during the 12 months preceding CR and SPC.

### Categorisation of patients

CR is not registered routinely in Danish health registers. Therefore, patients with CR were identified in the Danish nationwide health registries through validated register-based algorithms. Algorithms to identify patients diagnosed with recurrence from colorectal, breast and bladder cancer have been described in detail elsewhere [26–28]. The algorithms for malignant melanoma, endometrial and ovarian cancer performed similarly well, and papers on these are currently being prepared for publication. However, the algorithm for lung cancer has not yet been validated. In brief, patients were considered to have been successfully treated for cancer if no metastasis had been recorded at the time of the primary cancer diagnosis, and if procedural codes for curative intent cancer treatment had been recorded and followed by a period with no evidence of ongoing disease. Indicators of CR were based on malignant diagnosis codes, cancer-related procedure codes and pathology test results. The algorithms yielded sensitivities of 85–97% and specificities of 90–99%. The agreement between the recurrence dates generated by the algorithms and the gold standard, which was estimated by Lin’s concordance correlation coefficient [29], ranged from 0.964 to 0.996, which is considered as almost perfect [30].

In the present study, patients were categorised with CR if an indicator of recurrence was identified at any time from the first date after the required disease-free period until 31 December 2016. Patients were categorised with an SPC if registered with an ICD-10 code of C00-C96, except for C76-C80 (malignant neoplasms of ill-defined, secondary and unspecified sites) and C44 (other malignant neoplasms of skin) in the Danish Cancer Registry in the same time span. In case of both CR and SPC, these patients were categorised according to the first occurring event. If the two events occurred at the same date, these patients were excluded (*n* = 74).

Patients were considered in remission in the absence of CR and SPC or until the date of diagnosis of a subsequent cancer event (CR or SPC).

### Matching of cases

Patients with CR or SPC were matched 1:5 with patients in remission with same sex, primary cancer and comorbidity burden and belonging to the same age group as the CR or SPC case. Matching was performed using incidence density sampling. Comorbidity was defined according to the Charlson Comorbidity Index (CCI) [31], excluding cancer, on the basis of diagnosis codes from hospital contacts registered in the Danish National Patient Registry [32] during the 10-year period before the date of completed primary cancer treatment. CCI scores were categorised into three groups: “None” (score 0), “Low” (score 1–2) and “High” (score ≥ 3). Age was categorised into four groups on the basis of age at primary cancer diagnosis: “18–49 years”, “50–64 years”, “65–79 years” and “≥ 80 years”.

An index date was assigned to all patients. For the cases, the index date was defined as the date of CR or SPC. For the matched population, the index date was defined as the date occurring after the same number of days from the first date in remission until the diagnosis date of CR or SPC in their matched case. This was done to ensure the same follow-up period for patients with a subsequent cancer event and patients in remission. In the event of less than five matches, both the case and their matched patients were excluded (*n* = 134, hereof 37 cases) to obtain a balanced design.

### Defining the exposure

The exposure was defined as: “Cancer recurrence”, “Second primary cancer” or “In remission”. Patients in remission served as comparison population for patients with CR and for patients with SPC.

### Defining the outcome variable

Monthly rates of contacts in general practice and at hospitals in the twelve months preceding the index date were analysed. Data regarding contacts in general practice, including daytime face-to-face contacts, email consultations and telephone consultations, was obtained from the Danish National Health Service Register [33], which holds information on all contacts and procedures in primary care for reimbursement purposes. Hospital contacts included number of hospital admissions and outpatient visits (including specialist care and emergency department visits) registered in the Danish National Patient Register [32], which holds information on all contacts and procedures undertaken at somatic hospitals in Denmark.

### Other variables

Other variables in this study were sex, age, comorbidity, primary cancer disease, educational status, marital status and time since completion of primary cancer treatment. New age- and comorbidity-based groups were defined from age at index date and CCI at “first date of analysis” (i.e. the first date in the first month included in the analysis of activities in general practice and at hospitals) according to the patients with CR and SPC. Patients in remission were assigned to the same age and comorbidity group as their matched CR or SPC patient. Age and comorbidity groups were categorised as described under “Matching of cases” above (i.e. four age groups and three comorbidity groups).

Information on educational level and marital status was obtained from Statistics Denmark [34]. Educational level was categorised based on the highest attained education according to the International Standard Classification of Education (ISCED) [35] into: “Short” (levels I-II), “Medium” (levels III-IV) and “Long” (levels V-VI). Patients with no information on educational level (*n* = 907) were registered with “Short” as these persons are most often uneducated [36]. Marital status in the year prior to index date was categorised into: “Living alone” and “Married/cohabitating”. In case of missing values, information from five years back was obtained. Patients with no registrations five years back (*n* = 24) were classified as “Married/cohabitating”. The time period from completion of primary cancer treatment until index date was categorised into: “0–6 months”, “6–12 months”, “12–24 months”, “24–36 months”, “36–48 months” and “≥ 48 months”.

### Statistical analysis

Patients were matched using the Stata command *sttocc*. A negative binomial regression model applying cluster robust variance estimation to account for possible cluster effects at patient level was used to calculate a contact rate ratio (CRR) (i.e. incidence rate ratio) to enable comparison of monthly rates of healthcare contacts between patients with CR or SPC and patients in remission in the 12 months preceding the index date. If the index date occurred less than 13 months after ended primary cancer treatment, only contacts occurring later than one month after completion of cancer treatment were analysed.

Analyses were stratified by sex as differences in healthcare use have previously been documented to depend on sex [21, 22]. Analyses were further stratified on age group (defined at index date) and comorbidity status (calculated at “first date of analysis”). All analyses were performed as crude analyses and adjusted for confounding factors (educational level, marital status, time since completion of primary cancer treatment, primary cancer disease, age at index date and CCI score calculated on “first date of analysis”). Adjustments for age were done using restricted cubic splines with six knots according to Harrell’s recommended percentiles [37].

Crude rates of contacts were displayed using histograms with 95% confidence intervals (CI). Adjusted CRRs with 95% CIs were displayed using scatter plots. As patients naturally have very high frequencies of healthcare contacts in the month leading up to a diagnosis, we chose to omit the CRR estimate in the last month to illuminate the months of interest for the present study, i.e. the 2–12 months before the index date.

We undertook sensitivity analyses to test the robustness of the main analyses. Firstly, we excluded patients who died within 90 days after the index date. Secondly, we excluded patients diagnosed with SPC or CR within 180 days after the index date. Thirdly, we omitted all lung cancer patients since the algorithm to identify patients with recurrence of lung cancer had not been validated. Fourthly, we omitted patients with an index date of less than 13 months from the final date of treatment for the primary cancer to rule out that the observed activity originated from activity related to the first primary cancer disease. Lastly, we excluded patients with any of the above mentioned conditions in one sensitivity analysis.

A statistical level of *p* ≤ 0.05 was considered statistically significant in all analyses. Data were analysed using Stata® statistical software, version 15 (StataCorp LP, College Station, TX, USA).

## Results

**Fig. 1:**
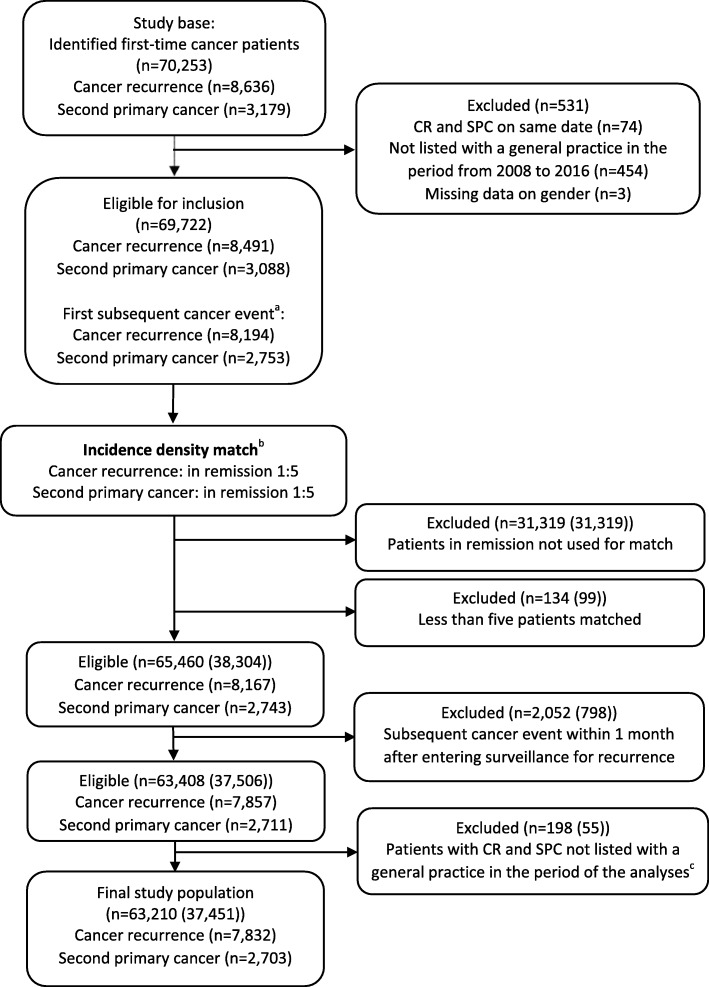
Flowchart of study population. ^a^Patients with both cancer recurrence and second primary cancer were categorised according to the first occurring event. bPatients matched on sex, age, comorbidity burden and primary cancer type. In the boxes below, the total number of observations is listed. In brackets, the number of unique patients is stated as patients in remission may serve as comparison subjects for more than one case. ^c^Matched comparison patients in remission were also excluded

The population eligible for inclusion consisted of 69,722 patients, hereof 8491 (12.2%) with CR and 3088 (4.4%) with SPC (Fig. 1). A total of 37,451 patients were included in the study after incidence density matching and exclusions. This population comprised 63,210 observations, hereof 7832 with CR, 2703 with SPC and 52,675 matched observations in continued complete remission. Patients with CR were younger and had higher levels of comorbidity compared to patients with SPC (Table 1). CR occurred closer in time to the primary cancer compared to SPC, and both CR and SPC occurred earlier in men compared to women. SPC diagnoses stratified on sex are presented in the additional files (Additional file 1).

**Table 1:** Study population characteristics^a^, stratified on sex and subsequent cancer event

	Women, n (%)						Men, n (%)					
	Cancer recurrence		Second primary cancer		In remission		Cancer recurrence		Second primary cancer		In remission	
Total population	5072	(100)	1811	(100)	34,415	(100)	2760	(100)	892	(100)	18,260	(100)
Age groups^b^												
18–49 years	624	(12.3)	85	(4.7)	3545	(10.3)	179	(6.5)	10	(1.1)	945	(5.2)
50–64 years	1556	(30.7)	461	(25.5)	10,085	(29.3)	728	(26.4)	144	(16.1)	4360	(23.9)
65–79 years	2188	(43.1)	972	(53.7)	15,800	(45.9)	1509	(54.7)	569	(63.8)	10,390	(56.9)
80+ years	704	(13.9)	293	(16.2)	4985	(14.5)	344	(12.5)	169	(18.9)	2565	(14.0)
Comorbidity^c^												
No	3045	(60.0)	1128	(62.3)	20,865	(60.6)	1292	(46.8)	509	(57.1)	9005	(49.3)
Low	1103	(21.7)	466	(25.7)	7845	(22.8)	758	(27.5)	272	(30.5)	5150	(28.2)
High	924	(18.2)	217	(12.0)	5705	(16.6)	710	(25.7)	111	(12.4)	4105	(22.5)
Primary cancer												
Bladder	103	(2.0)	19	(1.0)	610	(1.8)	281	(10.2)	72	(8.1)	1765	(9.7)
Malignant melanoma	467	(9.2)	197	(10.9)	3320	(9.6)	637	(23.1)	309	(34.6)	4730	(25.9)
Lung	400	(7.9)	64	(3.5)	2320	(6.7)	465	(16.8)	96	(10.8)	2805	(15.4)
Colorectal	1001	(19.7)	286	(15.8)	6435	(18.7)	1377	(49.9)	415	(46.5)	8960	(49.1)
Ovarian/endometrial	815	(16.1)	237	(13.1)	5260	(15.3)						
Breast	2286	(45.1)	1008	(55.7)	16,470	(47.9)						
Educational level^d^												
Short	2166	(42.7)	799	(44.1)	13,855	(40.3)	980	(35.5)	308	(34.5)	5966	(32.7)
Medium	1961	(38.7)	704	(38.9)	13,603	(39.5)	1390	(50.4)	453	(50.8)	9472	(51.9)
Long	945	(18.6)	308	(17.0)	6957	(20.2)	390	(14.1)	131	(14.7)	2822	(15.5)
Marital status^d^												
Cohabitating	2773	(54.7)	1007	(55.6)	19,147	(55.6)	1931	(70.0)	646	(72.4)	12,864	(70.4)
Living alone	2299	(45.3)	804	(44.4)	15,268	(44.4)	829	(30.0)	246	(27.6)	5396	(29.6)
Months since primary cancer^e^, median (IQR^f^)	16 (7;34)		32 (16;53)		20 (9;40)		11 (6;24)		26 (13;45)		14 (7;30)	

^a^All patients diagnosed with a primary cancer between 2008 and 2016 and followed until 31 December 2016. Patients with cancer recurrence and second primary cancer were matched 1:5 with patients in complete cancer remission

^b^Age at the time of cancer recurrence and second primary cancer, i.e. the index date

^c^Charlson Comorbidity Index score calculated on the first day of analysis, devided into 0 (none), 1–2 (low), 3+ (high)

^d^Educational level and marital status defined at the time of cancer recurrence and second primary cancer, i.e. the index date

^e^Time from final primary cancer treatment to a diagnosis of cancer recurrence or second primary cancer and the corresponding event date assigned to patients in remission, reported as median and interquartile range

^f^IQR: Interquartile range

### Healthcare utilisation in general practice and hospital

**Fig. 2:**
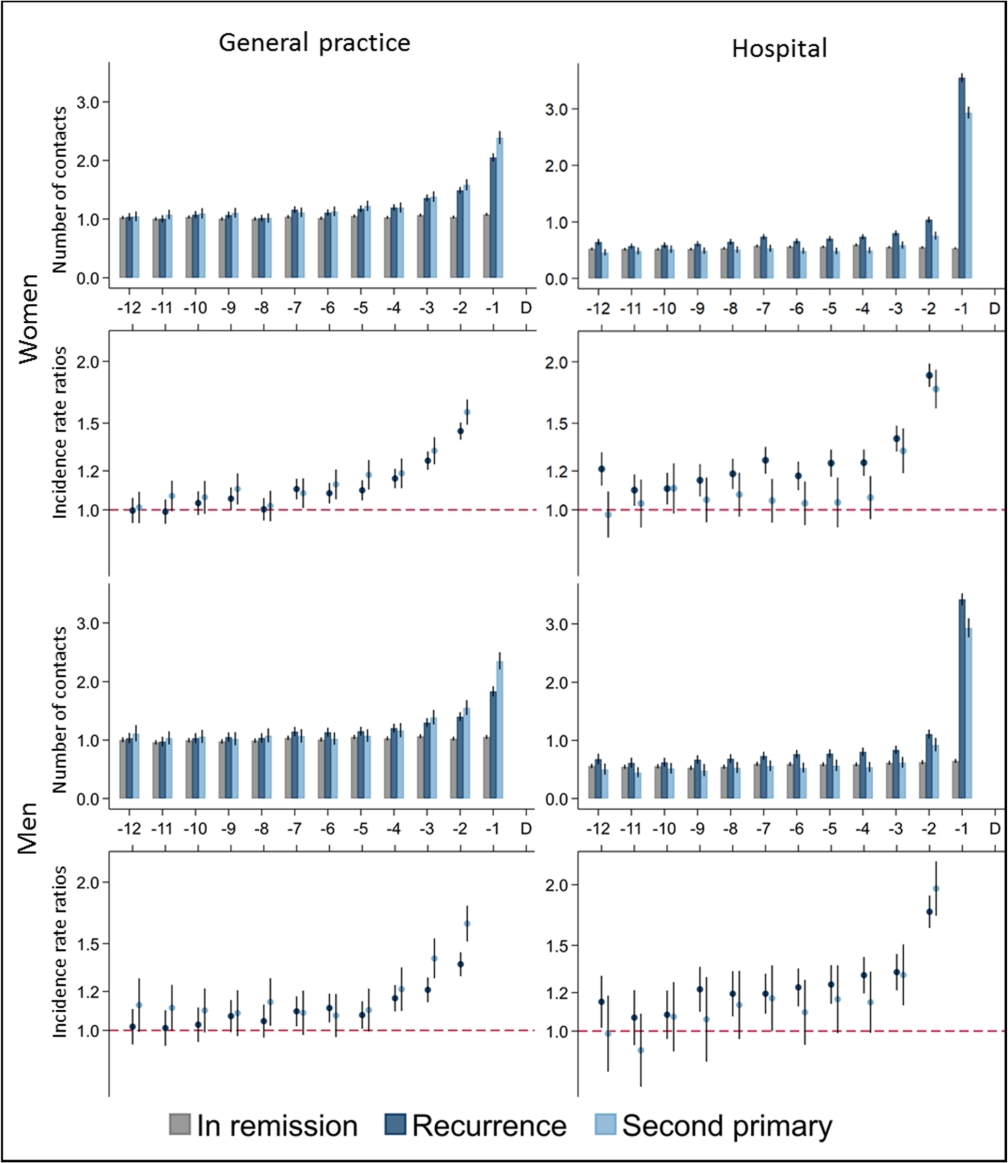
Number of contacts, stratified on sex and healthcare setting. Number of contacts are presented as crude rates of mean number of contacts per month. Incidence rate ratios were adjusted for age, comorbidity, educational level, marital status, primary cancer type and time since completion of primary cancer treatment. Patients in remission served as the reference group. Black lines represent 95% confidence intervals

Women had an average of one contact per month in general practice 12 months before the index date, irrespective of exposure group (Fig. 2). The contact rates in general practice increased from 10 and 11 months before the index date and onwards in women with CR and SPC, respectively, although these figures were only statistically significant when compared to women in remission from 7 months before the index date and onwards; CRRs increased from 1.10 (95% CI: 1.05–1.15) to 1.45 (95% CI: 1.39–1.50) in women with CR and from 1.08 (95% CI: 1.01–1.16) to 1.58 (95% CI: 1.49–1.67) in women with SPC. Findings were similar for the male population.

Women with CR had statistically significantly more hospital contacts throughout the entire year compared to women in remission; the CRR was 1.21 (95% CI: 1.12–1.31) at 12 months before CR and 1.88 (95% CI: 1.78–1.98) at 2 months before CR. The differences were less pronounced for women with SPC, and a similar pattern was seen in the male population.

As expected, the CRR was very high in the last month; the CRR in women was 7.16 (95% CI: 6.93–7.41) for hospital contacts and 1.93 (95% CI: 1.86–2.00) for general practice contacts in the last month before CR compared to women in remission (data not shown).

### Healthcare utilisation and age

Women and men had similar patterns of increasing number of contacts in general practice before CR and SPC across all age groups. However, in the age group 65–79 years, men with SPC had statistically significantly more contacts in general practice almost throughout the entire year compared to men in remission. For example, the CRRs were 1.22 (95% CI: 1.07–1.39) at 11 months and 1.18 (95% CI: 1.04–1.34) at 10 months before SPC. The difference in number of hospital contacts for women with CR compared to women in remission was most pronounced in the two youngest age groups; women with CR aged 50–64 years had 44% more hospital contacts at 12 months before the index date compared to the matched women in remission (95% CI: 1.26–1.65) and had statistically significantly more hospital contacts throughout the entire year. The findings were similar in men, although less pronounced. A graphical presentation of contact rates and CRRs stratified on sex, healthcare setting and age groups are shown in the additional files (Additional files 2 and 3).

### Healthcare utilisation and comorbidity status

Patients with CR and SPC had very similar contact patterns in general practice within each comorbidity group; this was seen in both women and men. Women with CR and no comorbidity had statistically significantly more hospital contacts compared to women in remission throughout the entire year; the CRRs ranged from 1.59 (95% CI: 1.05–1.27) to 2.13 (95% CI: 1.99–2.29). Contact patterns were similar in women with low comorbidity, although less pronounced. Hospital contact patterns were similar for men, although the differences were less pronounced. A graphical presentation of contact rates and CRRs stratified on sex, healthcare setting and comorbidity groups are shown in the additional files (Additional files 4 and 5).

### Sensitivity analyses

The results from the four sensitivity analyses (see detailed description in “Statistical analyses”) showed the same overall pattern for sex as that shown in the main analyses. When all sensitivity analyses were combined into one analysis, the contact rates in the last month decreased slightly, but the CRR remained largely unchanged. A graphical presentation of all sensitivity analyses are shown in the additional files (Additional files 6, 7, 8 and 9).

## Discussion

### Main findings

This study of more than 63,000 curatively treated cancer patients in remission showed that cancer survivors developing CR or SPC began to increase their contact frequency in general practice from seven months before a subsequent cancer diagnosis and at hospitals from up to one year before a subsequent cancer diagnosis. These changes were more pronounced in patients with recurrence of cancer, younger patients and patients with no comorbidity.

### Strengths and limitations

To our knowledge, this study is the first to investigate healthcare use preceding a diagnosis of CR and SPC in a nationwide setting on the basis of prospectively collected data from registers with high completeness and accuracy [25, 32, 33]. Performing epidemiological population-based register studies of patients with CR is a challenging task because remission and CR are not routinely recorded outside of clinical trials [38]. However, newly developed algorithms allowed us to identify patients with CR in Denmark [26–28]. The study included patients diagnosed with primary cancer over a nine-year period and up to nine years of follow-up; this resulted in as many as 7832 patients with CR and 2703 patients with SPC and enabled us to conduct sex-, age- and comorbidity-stratified analyses with high statistical precision.

The main limitation of the study is that the sensitivity of the algorithms used to identify patients with CR ranged from 85 to 97% and the specificity from 93 to 99% [26–28]. This may have misclassified a minor proportion of CRs as being in continued remission. Such misclassification is likely to have underestimated the differences in healthcare use between patients with CR and patients in remission and thus have lowered the CRR estimates. The agreement between the recurrence dates estimated by the algorithms and the gold standards was high [27–30]. Nevertheless, some recurrence dates were estimated by the algorithm to occur later in time than the true recurrence dates. This may have overestimated the number of pre-diagnostic contacts because contacts related to CR treatment would then be categorised as pre-diagnostic activity. However, this would be counterbalanced by an underestimated number of pre-diagnostic contacts in patients for whom the CR date was estimated to be earlier than the true CR date. Hence, misclassifications of the recurrence dates are not considered to have altered the results.

Changes in contact rates were considered a proxy for symptom presentation of CR and SPC. We included all contacts to general practice and all hospital contacts, and some of these contacts could concern other health problems. Still, we aimed to eliminate confounding by matching on primary cancer disease, age group and CCI score and by adjusting for factors known to affect healthcare use. Finally, we conducted sensitivity analyses excluding some groups of patients, e.g. patients who died within 90 days after the index date (to eliminate activity related to death), patients diagnosed with SPC or CR within 180 days after the index date (to eliminate activity related to a third cancer diagnosis), and patients with an index date of less than 13 months from the final date of treatment for the primary cancer (to eliminate activity originating from the first primary cancer disease). Increased healthcare activity remained for 7 months in general practice and for up to 12 months in hospitals; this was seen even when all four sensitivity analyses were included in one analysis, which suggests that the increase in activity was related to CR and SPC.

More than 65% of the female study population consisted of survivors from colorectal cancer and breast cancer, and almost 50% of the male study population were survivors from colorectal cancer. Although we adjusted for primary cancer disease in the analyses, the patients with breast or colorectal cancers may have dominated the results over smaller populations with bladder or lung cancers. High-definition analyses stratified on primary cancer disease are thus warranted, but these were outside the scope of this study.

### Comparison with relevant literature

We found increasing contact frequency in general practice, starting from eleven months before CR and SPC. Other studies have reported similar findings for a first primary cancer diagnosis [21, 22, 39]. Thus, GPs seem to be as involved in the detection of CR and SPC as in the diagnosis of a first primary cancer.

In the present study, we assessed healthcare activity related to CR and SPC as the increased activity in these patients compared to the activity in patients with no second cancer event, which is illustrated by the contact rates and CRRs in Fig. 2. Augestad et al. [23] found a 29% probability of CR after initiation of diagnostic work-up based on suspicion of colorectal CR. This increased base consultation rate in patients in remission infers that an elevated activity in patients with a second cancer event is more difficult to detect, why the true onset of elevated activity might be at earlier point in time than illustrated by the contact rates and the CRRs in Fig. 2.

Women tended to have higher CRRs in hospitals prior to CR compared to SPC. This difference was less pronounced in men, which may be explained by the fact that, in this study, 17% of the SPCs in women were breast cancers. Breast cancer is defined as an “easy to diagnose” type of cancer, i.e. a cancer presenting with clear symptoms, which has been associated with fewer contacts prior to diagnosis [40].

The CRRs were most increased in younger and non-comorbid cancer survivors. Lyratzopoulos et al. [40] attributed similar findings for first-time primary cancers to a poorer understanding of cancer symptoms in younger patients. Thus, an altered focus on symptom presentation to ensure timely detection in younger patients may be warranted. Patients with comorbidity had more hospital contacts compared to patients with no comorbidity. This group may address both issues related to comorbid disorders and symptoms of subsequent cancer events in a consultation scheduled exclusively for a chronic disorder. This could have caused the lower differences in contact rates in this group compared to the reference population [41]. Hence, we can neither prove nor reject that patients with comorbid disorders may also have presented CR- and SPC-related symptoms at an earlier point in time. The analyses stratified on age groups and comorbidity revealed a need for increased focus on younger patients, and they indicated that symptom presentation of CR and SPC may occur earlier in time than illustrated by the analyses stratified on sex alone.

### Interpretation and implications

We found increased healthcare contacts in both general practice and at hospitals from up to one year before a subsequent cancer diagnosis. This finding indicates a potential to diagnose CR and SPC at an earlier point in time and to improve both the organisation of follow-up and the coordination across sector boundaries.

The data for this study originates from a time when cancer follow-up was organised in specialised hospital departments. Given the median time of 11 months to CR and of 32 months to SPC, patients were most likely actively followed at the time of CR and SPC. We demonstrated increasing activity in general practice prior to a subsequent cancer event, which could indicate that patients found the GP more accessible despite direct access to specialised hospital departments. This is in line with a study by Grunfeld et al. [17], who reported that most breast cancer recurrences were detected as interval events and that women presented first in general practice despite hospital-based follow-up. Furthermore, studies in gynaecological cancers [42–44], malignant melanoma [45] and colorectal cancers [19] found between 42 and 72% of CRs to be diagnosed as interval events outside scheduled follow-up visits and to be symptomatic at diagnosis. Gilbert et al. [46] found 67% of lung CRs to be detected by the GP. This indicates involvement of general practice and supports that the increased contact rates in general practice found in the present study were related to detection of CR and SPC.

If higher use of healthcare before CR or SPC can be regarded as an indication of increased symptoms caused by the cancer, the findings of this study indicate that prolonged diagnostic pathways prevail in the diagnosis of subsequent cancer events. This is similar to the conclusions made in other studies on first-time primary cancers [21, 22]. Recent studies exploring frequent vs. less frequent diagnostic testing as part of follow-up found no impact on CR detection, time to CR detection and (overall or cancer-specific) mortality [3, 47]. Hence, additional approaches to follow-up testing must be considered to ensure more timely detection of CR and SPC. We need a better understanding of the symptom presentation in CR and SPC, and how patients and healthcare professionals react to new symptoms. Furthermore, considerations should be made on the best use of the limited resources in the healthcare system, and risk stratified follow-up pathways should be considered [48]. Studies have reported a false positive rate of 71% in tests, which all raised suspicion of CR [23], and a probability of 87% for at least one false positive test within five years of follow-up [49]. The authors of these studies have suggested risk-tailored surveillance to ensure more cost-effective follow-up, a better balance between harms and benefits from diagnostic investigations and improved pathways for patients.

We found more hospital contacts for patients with CR compared to patients with SPC, primarily for women. This may be due to clearer symptom presentation in SPC and prompt referral to well-organised cancer treatment pathways for primary cancers. Cancer treatment pathways for recurrence do not exist in Denmark, and uniform pathways are challenging to describe as the relevant actions vary according to whether a cancer recurs locally or in a distant site and depending on the state of the patient. However, the increased healthcare activity for up to one year in both general practice and hospitals prior to a diagnosis of CR indicates that optimised diagnostic pathways for CR are warranted. Although some of the increased pre-diagnostic activity could be related to poorer prognosis, the results still indicate that a window of opportunity for earlier diagnosis of CR and SPC exists in both general practice and in hospitals.

Our findings suggest that the current organisation of follow-up is suboptimal as patients with CR and SPC increased their use of both general practice and hospital-based services long before the subsequent CR or SPC was diagnosed. Diagnosing at an earlier point in time requires the patient to present with symptoms earlier, the healthcare provider to react more adequately and faster when patients present with symptoms, and it may also require organisational changes in the healthcare system [50]. Patient delay, i.e. the time from when the patient assesses a symptom, decides to seek healthcare and schedules an appointment, was not assessed in this study, and the window of opportunity for more timely diagnosis may open at an earlier point in time than illustrated by the increased healthcare contacts. Suboptimal coordination and communication between general practice and hospitals is a well-known challenge in cancer survivorship care [51]. Difficulties in the transition from hospitals to general practice and unclear responsibility areas remain focal points for healthcare professionals and researchers [12]. Improved communication between general practice and hospitals is important, irrespective of follow-up model [16], and pathways for communication should be formalised, clearly defined and easily accessible [12]. Formal involvement of GPs in cancer follow-up could be a benefit as we found that the GP has regular contact with cancer survivors and seems to be involved in the detection of subsequent cancer events. Additionally, it could be more convenient for the patients [52]. However, it is important to provide direct access for the GP to relevant diagnostic investigations and to specialist care when conducting GP-led cancer follow-up [12, 16]. Healthcare professionals and planners should decide on models for cancer survivorship care, define the roles of general practice and hospitals, including areas of responsibility, and advocate for meaningful resource allocation to ensure the best possible future organisation of high-quality follow-up programmes, including well-organised diagnostic pathways for CR and SPC [1].

## Conclusion

The present study shows that most patients are increasingly in contact with both general practice and hospitals for several months before the detection of a recurring cancer or second primary cancer. We demonstrated that a window of opportunity for more timely diagnosis of subsequent cancer is open from seven months earlier in general practice and from up to one year earlier in the hospital setting. Our findings imply that closer collaboration between healthcare sectors is warranted.

## Supplementary information

**Additional file 1.** Second primary cancers diagnosed between 2008 and 2016, stratified on sex.

**Additional file 2.** Number of contacts in women, stratified on healthcare setting and age.

**Additional file 3.** Number of contacts in men, stratified on healthcare setting and age.

**Additional file 4.** Number of contacts in women, stratified on healthcare setting and comorbidity burden.

**Additional file 5.** Number of contacts in men, stratified on healthcare setting and comorbidity burden.

**Additional file 6.** Sensitivity analysis of number of contacts to general practice in women.

**Additional file 7.** Sensitivity analysis of number of contacts to general practice in men.

**Additional file 8.** Sensitivity analysis of number of hospital contacts in women.

**Additional file 9.** Sensitivity analysis of number of hospital contacts in men.

## Data Availability

The data supporting the findings of this study is stored and maintained electronically at Statistics Denmark. The data is only accessible via a secured virtual private network (VPN) and only by approved collaborative partners. The data is not publicly available due to the Danish data protection legislation as the data contains information that could compromise the privacy of the research participants.

## Abbreviations

CCI: Charlson Comorbidity Index
CI: Confidence interval
CR: Cancer recurrence
CRR: Contact rate ratio
GP: General practitioner
ICD-10: International Classification of Diseases, 10th revision
ISCED: International Standard Classification of Education

## References

[1] Rubin G, Berendsen A, Crawford SM, Dommett R, Earle C, Emery J (2015). The expanding role of primary care in cancer control. Lancet Oncol.

[2] Ignatov A, Eggemann H, Burger E, Ignatov T (2018). Patterns of breast cancer relapse in accordance to biological subtype. J Cancer Res Clin Oncol.

[3] Wille-Jorgensen P, Syk I, Smedh K, Laurberg S, Nielsen DT, Petersen SH (2018). Effect of more vs less frequent follow-up testing on overall and colorectal cancer-specific mortality in patients with stage II or III colorectal cancer: the COLOFOL randomized clinical trial. JAMA.

[4] Hassett MJ, Uno H, Cronin AM, Carroll NM, Hornbrook MC, Ritzwoller D (2017). Detecting lung and colorectal cancer recurrence using structured clinical/administrative data to enable outcomes research and population health management. Med Care.

[5] Zattoni F, Morlacco A, Nehra A, Frank I, Boorjian SA, Thapa P (2018). Vaginal cuff recurrence after radical cystectomy: an under - studied site of bladder cancer relapse. Int Braz J Urol.

[6] Soerjomataram I, Coebergh JW (2009). Epidemiology of multiple primary cancers. Methods Mol Biol.

[7] Gelli M, Huguenin JFL, de Baere T, Benhaim L, Mariani A, Boige V (2018). Peritoneal and extraperitoneal relapse after previous curative treatment of peritoneal metastases from colorectal cancer: what survival can we expect?. Eur J Cancer.

[8] Taylor JM, Spiess PE, Kassouf W, Munsell MF, Kamat AM, Dinney CP (2010). Management of urethral recurrence after orthotopic urinary diversion. BJU Int.

[9] Hung JJ, Hsu WH, Hsieh CC, Huang BS, Huang MH, Liu JS (2009). Post-recurrence survival in completely resected stage I non-small cell lung cancer with local recurrence. Thorax.

[10] Neal RD, Tharmanathan P, France B, Din NU, Cotton S, Fallon-Ferguson J (2015). Is increased time to diagnosis and treatment in symptomatic cancer associated with poorer outcomes? Systematic review. Br J Cancer.

[11] Torring ML, Murchie P, Hamilton W, Vedsted P, Esteva M, Lautrup M (2017). Evidence of advanced stage colorectal cancer with longer diagnostic intervals: a pooled analysis of seven primary care cohorts comprising 11 720 patients in five countries. Br J Cancer.

[12] Nekhlyudov L, O'malley DM, Hudson SV (2017). Integrating primary care providers in the care of cancer survivors: gaps in evidence and future opportunities. Lancet Oncol.

[13] McCabe MS, Partridge AH, Grunfeld E, Hudson MM (2013). Risk-based health care, the cancer survivor, the oncologist, and the primary care physician. Semin Oncol.

[14] Meiklejohn JA, Mimery A, Martin JH, Bailie R, Garvey G, Walpole ET (2016). The role of the GP in follow-up cancer care: a systematic literature review. J Cancer Surviv.

[15] Lewis RA, Neal RD, Hendry M, France B, Williams NH, Russell D (2009). Patients’ and healthcare professionals' views of cancer follow-up: systematic review. Br J Gen Pract.

[16] Lewis RA, Neal RD, Williams NH, France B, Hendry M, Russell D (2009). Follow-up of cancer in primary care versus secondary care: systematic review. Br J Gen Pract.

[17] Grunfeld E, Mant D, Yudkin P, Adewuyi-Dalton R, Cole D, Stewart J (1996). Routine follow up of breast cancer in primary care: randomised trial. BMJ.

[18] Christensen NL, Dalton SO, Mellemgaard A, Christensen J, Kejs AMT, Rasmussen TR (2018). Assessing the pattern of recurrence in Danish stage I lung cancer patients in relation to the follow-up program: are we failing to identify patients with cerebral recurrence?. Acta Oncol.

[19] Duineveld LA, van Asselt KM, Bemelman WA, Smits AB, Tanis PJ, van Weert HC (2016). Symptomatic and asymptomatic colon cancer recurrence: a multicenter cohort study. Ann Fam Med.

[20] Brandenbarg D, Berendsen AJ, de Bock GH (2017). Patients’ expectations and preferences regarding cancer follow-up care. Maturitas.

[21] Christensen KG, Fenger-Gron M, Flarup KR, Vedsted P (2012). Use of general practice, diagnostic investigations and hospital services before and after cancer diagnosis - a population-based nationwide registry study of 127,000 incident adult cancer patients. BMC Health Serv Res.

[22] Hansen PL, Hjertholm P, Vedsted P (2015). Increased diagnostic activity in general practice during the year preceding colorectal cancer diagnosis. Int J Cancer.

[23] Augestad KM, Norum J, Rose J, Lindsetmo R-O (2014). A prospective analysis of false positive events in a national Colon Cancer surveillance program. BMC Health Serv Res.

[24] Pedersen KM, Andersen JS, Søndergaard J (2012). General practice and primary health care in Denmark. J Am Board Fam Med.

[25] Gjerstorff ML (2011). The Danish cancer registry. Scand J Public Health.

[26] Lash TL, Riis AH, Ostenfeld EB, Erichsen R, Vyberg M, Thorlacius-Ussing O (2015). A validated algorithm to ascertain colorectal cancer recurrence using registry resources in Denmark. Int J Cancer.

[27] Rasmussen LA, Jensen H, Virgilsen LF, Jensen JB, Vedsted P (2018). A validated algorithm to identify recurrence of bladder cancer: a register-based study in Denmark. Clin Epidemiol.

[28] Rasmussen LA, Jensen H, Virgilsen LF, Thorsen LBJ, Offersen BV, Vedsted P (2019). A validated algorithm for register-based identification of patients with recurrence of breast cancer in Denmark – based on Danish Breast Cancer Group (DBCG) data. Cancer Epidemiol.

[29] Lin LI (1989). A concordance correlation coefficient to evaluate reproducibility. Biometrics.

[30] McBride GB (2005). A proposal for strength of agreement criteria for Lin's concordance correlation coefficient.

[31] Quan H, Li B, Couris CM, Fushimi K, Graham P, Hider P (2011). Updating and validating the Charlson comorbidity index and score for risk adjustment in hospital discharge abstracts using data from 6 countries. Am J Epidemiol.

[32] Schmidt M, Schmidt SA, Sandegaard JL, Ehrenstein V, Pedersen L, Sorensen HT (2015). The Danish National Patient Registry: a review of content, data quality, and research potential. Clin Epidemiol.

[33] Andersen JS, Olivarius Nde F, Krasnik A (2011). The Danish National Health Service Register. Scand J Public Health.

[34] Statistics Denmark: Documentation of statistics. Available at: https://www.dst.dk/en/Statistik/dokumentation/documentationofstatistics. Accessed 20 April 2019.

[35] UNESCO Institute for Statistics (2012). International standard classification of education ISCED 2011.

[36] Statistics Denmark. Uddannelsesstatistikkens manual [Manual of educational statistics]. Copenhagen: Statistics Denmark; 2016.

[37] Harrell FE (2001). Regression modeling strategies: with applications to linear models LR, and survival analysis.

[38] Warren JL, Yabroff KR (2015). Challenges and opportunities in measuring cancer recurrence in the United States. J Natl Cancer Inst.

[39] Jensen H, Vedsted P, Moller H (2018). Consultation frequency in general practice before cancer diagnosis in relation to the patient's usual consultation pattern: a population-based study. Cancer Epidemiol.

[40] Lyratzopoulos G, Neal RD, Barbiere JM, Rubin GP, Abel GA (2012). Variation in number of general practitioner consultations before hospital referral for cancer: findings from the 2010 National Cancer Patient Experience Survey in England. Lancet Oncol.

[41] Heins M, Schellevis F, Rijken M, van der Hoek L, Korevaar J (2012). Determinants of increased primary health care use in cancer survivors. J Clin Oncol.

[42] Salani R, Backes FJ, Fung MFK, Holschneider CH, Parker LP, Bristow RE (2011). Posttreatment surveillance and diagnosis of recurrence in women with gynecologic malignancies: Society of Gynecologic Oncologists recommendations. Am J Obstet Gynecol.

[43] Nordin AJ (2006). Mode of detection of recurrent gynecological malignancy: does routine follow-up delay diagnosis and treatment?. Int J Gynecol Cancer.

[44] Jeppesen MM, Mogensen O, Hansen DG, Iachina M, Korsholm M, Jensen PT (2017). Detection of recurrence in early stage endometrial cancer – the role of symptoms and routine follow-up. Acta Oncol (Madr).

[45] Francken AB, Shaw HM, Accortt NA, Soong S-J, Hoekstra HJ, Thompson JF (2007). Detection of first relapse in cutaneous melanoma patients: implications for the formulation of evidence-based follow-up guidelines. Ann Surg Oncol.

[46] Gilbert S, Reid KR, Lam MY, Petsikas D (2000). Who should follow up lung cancer patients after operation?. Ann Thorac Surg.

[47] Snyder RA, Hu CY, Cuddy A, Francescatti AB, Schumacher JR, Van Loon K (2018). Association between intensity of posttreatment surveillance testing and detection of recurrence in patients with colorectal cancer. JAMA.

[48] Rasmussen LA, Jensen H, Virgilsen LF, Falborg AZ, Møller H, Vedsted P (2019). Time from incident primary cancer until recurrence or second primary cancer: risk factors and impact in general practice. Eur J Cancer Care (Engl).

[49] Augestad KM, Rose J, Crawshaw B, Cooper G, Delaney C (2014). Do the benefits outweigh the side effects of colorectal cancer surveillance? A systematic review. World J Gastrointest Oncol.

[50] Weller D, Vedsted P, Rubin G, Walter FM, Emery J, Scott S (2012). The Aarhus statement: improving design and reporting of studies on early cancer diagnosis. Br J Cancer.

[51] Grunfeld E, Earle CC (2010). The interface between primary and oncology specialty care: treatment through survivorship. J Natl Cancer Inst Monogr.

[52] Grunfeld E, Levine MN, Julian JA, Coyle D, Szechtman B, Mirsky D (2006). Randomized trial of long-term follow-up for early-stage breast cancer: a comparison of family physician versus specialist care. J Clin Oncol.

