# Supplementary material for: Healthcare utilisation in general practice and hospitals in the year preceding a diagnosis of cancer recurrence or second primary cancer: a population-based register study
Source: BMC Health Serv Res. 2019 Dec 5;19:941. doi: 10.1186/s12913-019-4757-y (PMC6896499; doi:10.1186/s12913-019-4757-y)
Supplement: Supplementary file 1 — Additional file 1. Second primary cancers diagnosed between 2008 and 2016, stratified on sex. [file 12913_2019_4757_MOESM1_ESM.pdf]

Additional file 1:

Second primary cancers diagnosed between 2008 and 2016, stratified on sex, n (%)

	Women		Men		Total	
Lip, oral cavity and pharynx	44	(2)	32	(4)	76	(3)
Oesphagus and stomach	46	(3)	36	(4)	82	(3)
Small intestine	10	(1)	5	(1)	15	(1)
Colon and rectum	327	(18)	115	(13)	442	(16)
Other digestive organs	90	(5)	38	(4)	128	(5)
Lung	318	(18)	144	(16)	462	(17)
Other respiratory and intrathoracic sites	9	(1)	21	(2)	30	(1)
Malignant melanoma	116	(6)	38	(4)	154	(6)
Mesothelial and soft tissue	30	(2)	15	(2)	45	(2)
Breast	298	(17)	*		298	(11)
Endometrium	99	(5)	-		99	(4)
Ovary	60	(3)	-		60	(2)
Other female genital organs	43	(2)	-		43	(2)
Male genital organs	-		252	(28)	252	(9)
Bladder	32	(2)	37	(4)	69	(3)
Other sites of the urinary tract	56	(3)	42	(5)	98	(4)
Central nervous system	35	(2)	17	(2)	52	(2)
Endocrine glands	29	(2)	7	(1)	36	(1)
Lymphoid, haematopoietic and related tissue	147	(8)	75	(8)	222	(8)
Other	22	(1)	18	(2)	40	(1)
Total	1,811	(100)	892	(100)	2,703	(100)

*Male breast cancer is reported in “Other”
