# Supplementary material for: Healthcare utilisation in general practice and hospitals in the year preceding a diagnosis of cancer recurrence or second primary cancer: a population-based register study
Source: BMC Health Serv Res. 2019 Dec 5;19:941. doi: 10.1186/s12913-019-4757-y (PMC6896499; doi:10.1186/s12913-019-4757-y)
Supplement: Supplementary file 3 — Additional file 3. Number of contacts in men, stratified on healthcare setting and age. [file 12913_2019_4757_MOESM3_ESM.pdf]

Additional file 3: Number of contacts in men, stratified on healthcare setting and age

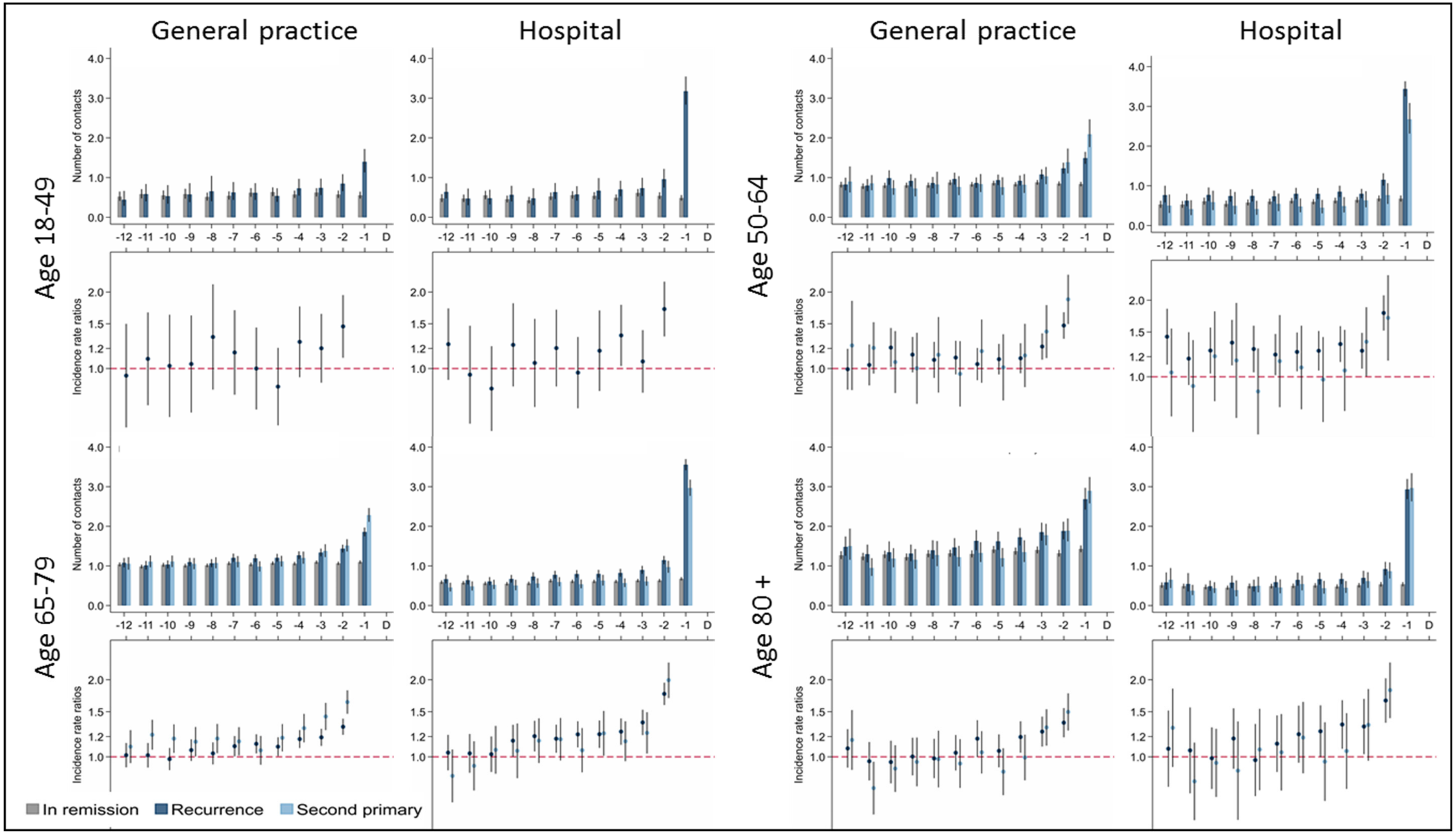

Number of contacts are presented as crude rates of mean number of contacts per month. Consultation rate ratios were adjusted for age, comorbidity, educational level, marital status, primary cancer type and time since completion of primary cancer treatment. Patients in remission served as the reference group. Black lines represent 95% confidence intervals. Estimates for men with second primary cancer and aged 18-49 years are omitted due to few observations to adhere to current data privacy protection legislation.
