# Supplementary material for: Healthcare utilisation in general practice and hospitals in the year preceding a diagnosis of cancer recurrence or second primary cancer: a population-based register study
Source: BMC Health Serv Res. 2019 Dec 5;19:941. doi: 10.1186/s12913-019-4757-y (PMC6896499; doi:10.1186/s12913-019-4757-y)
Supplement: Supplementary file 7 — Additional file 7. Sensitivity analysis of number of contacts to general practice in men. [file 12913_2019_4757_MOESM7_ESM.pdf]

Additional file 7: Sensitivity analysis of number of contacts to general practice in men

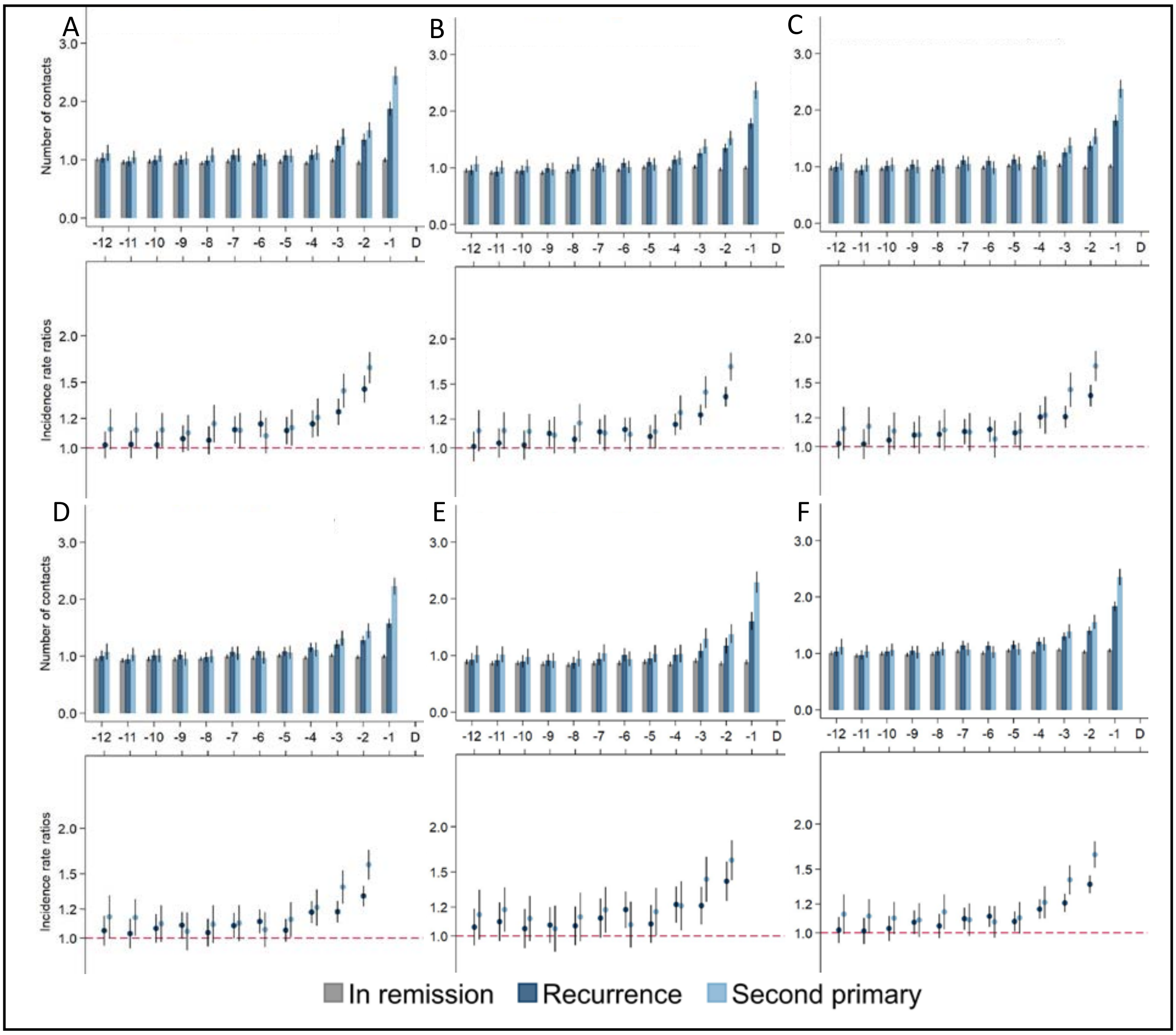

A. Excluding patients with cancer recurrence and second primary cancer less than 13 months after completion of primary cancer treatment and the matched patients in remission
B. Excluding all lung cancer patients
C. Excluding patients with cancer recurrence and second primary cancer with a third cancer event within 180 days after the index date and the matched patients in remission
D. Excluding patients with cancer recurrence and second primary cancer who died within 90 days after the index date and the matched patients in remission
E. Excluding all patients excluded in bullet A, B, C and D
F. No exclusions

Number of contacts are presented as crude rates of mean number of contacts per month. Consultation rate ratios were adjusted for age, comorbidity, educational level, marital status, primary cancer type and time since completion of primary cancer treatment. Patients in remission served as the reference group. Black lines represent 95% confidence intervals.
